# Suggested Novel Risk Factors for Swimming-Induced Pulmonary Edema: Asthma and Limited Open Water Swimming Experience

**DOI:** 10.1016/j.chest.2026.02.039

**Published:** 2026-03-26

**Authors:** Linda Kristiansson, Claudia Seiler, Annika Braman Eriksson, Josefin Sundh, Maria Hårdstedt

**Affiliations:** a School of Medical Sciences, Faculty of Medicine and Health, Örebro University, Örebro, Sweden; b Sandviken North Primary Health Care Center, Sandviken, Sweden; c Center for Research and Development, Uppsala University/Region Gävleborg, Gävle, Sweden; d Center for Clinical Research Dalarna, Uppsala University, Falun, Sweden; e Department of Anesthesiology and Intensive Care, Falun Hospital, Falun, Sweden; f Vansbro Primary Health Care Center, Vansbro, Sweden; g Department of Respiratory Medicine, Faculty of Medicine and Health, Örebro University, Örebro, Sweden

**Keywords:** asthma, heart diseases, hypertension, immersion pulmonary edema, respiratory tract infections, risk factor, sex, SIPE, swimming, swimming-induced pulmonary edema

## Abstract

**Background:**

Swimming-induced pulmonary edema (SIPE) has gained increasing attention in recent years. However, risk factors for SIPE remain incompletely understood, and existing data are primarily based on small-scale studies.

**Research Question:**

Which individual risk factors are associated with SIPE?

**Study Design and Methods:**

We conducted a case-control study based on Sweden’s largest open water swimming event, Vansbrosimningen. Data were obtained through interviews with individuals diagnosed with SIPE in 2017 to 2024 and through a web-based survey administered to control participants without SIPE. Individual factors associated with SIPE were identified by using multiple logistic regression.

**Results:**

A total of 258 cases diagnosed with SIPE and 2,117 control participants were included. A first logistic regression model identified female sex (OR, 5.3; 95% CI, 3.6-7.7), higher age (OR for age ≥ 61 years, 7.3; 95% CI, 3.8-14.2), hypertension (OR, 1.9; 95% CI, 1.2-3.2), heart disease (OR, 6.0; 95% CI, 2.5-14.2), asthma (OR, 1.7; 95% CI, 1.2-2.6), and low frequency of open water swimming during the current season (OR for never, 3.6; 95% CI, 2.4-5.4) as independently associated with SIPE. A second model found that previous respiratory symptoms during open water swimming (OR, 3.5; 95% CI, 2.3-5.2) and acute respiratory tract infection (OR, 10.2; 95% CI, 5.2-20.2) were also strongly associated with SIPE; however, the association with asthma failed statistical significance in this model.

**Interpretation:**

This study confirms previously reported individual risk factors for SIPE and immersion pulmonary edema: female sex, higher age, hypertension, heart disease, respiratory tract infection, and prior episodes. The data also suggest that asthma and limited experience of open water swimming are associated with an increased risk of SIPE. A comprehensive understanding of the risk factors for SIPE will improve safety during open water swimming and contribute to the development of medical guidelines for SIPE.

## Graphical Abstract

**Figure:**
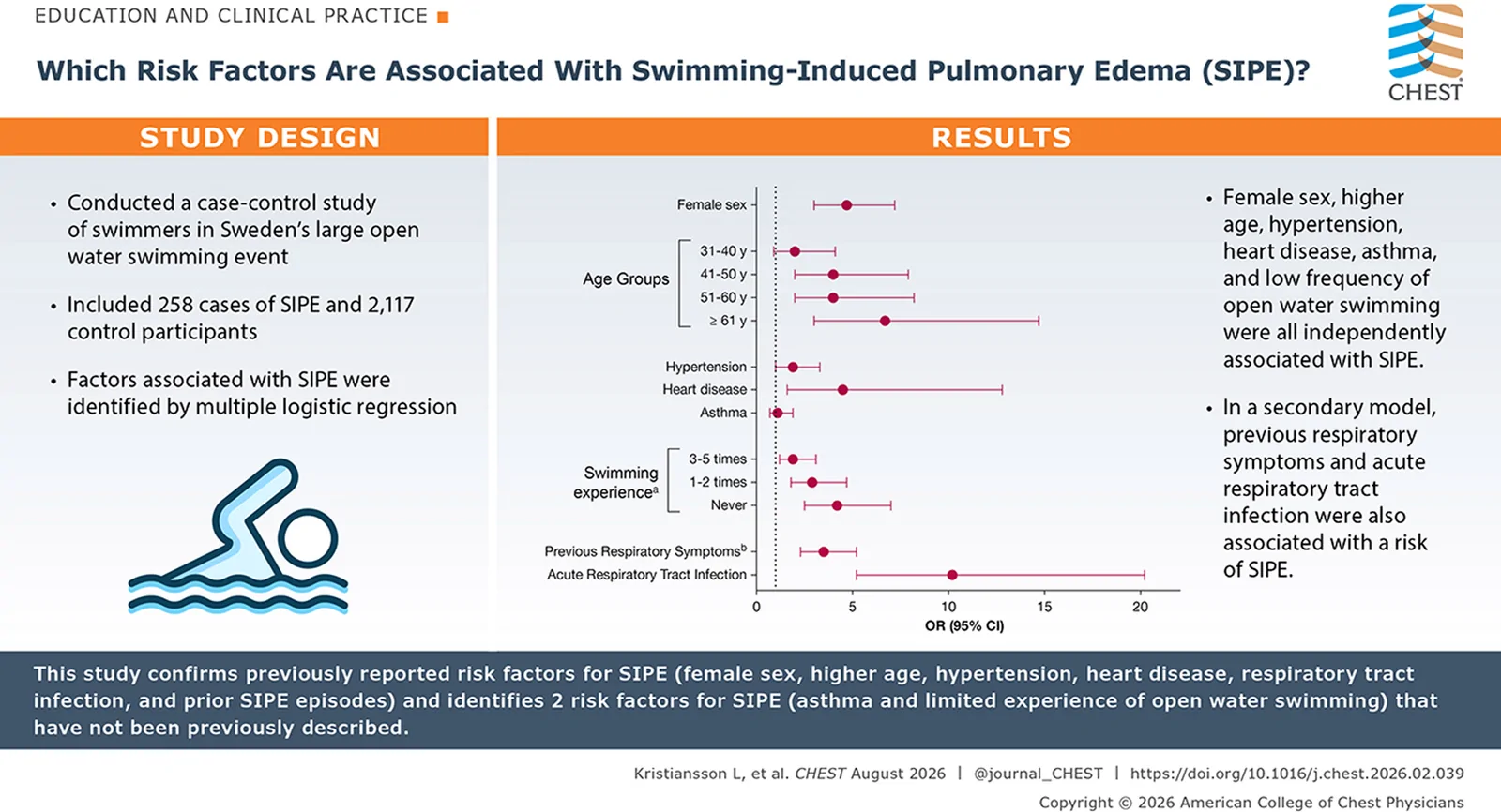

FOR EDITORIAL COMMENT, SEE PAGE 297

**Take-Home Points**

**Research Question:** Which individual risk factors are associated with swimming-induced pulmonary edema (SIPE)?

**Results:** Based on a large cohort of open water swimmers, we confirmed female sex, higher age, hypertension, heart disease, acute respiratory tract infection, and previous respiratory symptoms during open water swimming as risk factors for SIPE and further suggest that asthma and limited experience of open water swimming are associated with an increased risk of SIPE.

**Interpretation:** Two risk factors for SIPE are suggested here that have not previously been described in the literature: asthma and limited experience of open water swimming.

Swimming-induced pulmonary edema (SIPE) is a subgroup of immersion pulmonary edema (IPE) occasionally occurring during open water swimming.^1^ The symptoms of SIPE include cough, dyspnea, exhaustion, increased sputum, and hemoptysis.^2^

The pathophysiology of SIPE is not completely understood, although several potential risk factors have been discussed.^2^, ^3^, ^4^, ^5^ In our previous studies conducted during Sweden’s largest open water swimming event, Vansbrosimningen, SIPE was more frequently observed in female individuals and in those of older age, which is consistent with other studies of SIPE and IPE.^6^, ^7^, ^8^, ^9^, ^10^, ^11^, ^12^ We also observed a higher prevalence of asthma in patients with SIPE than in the general population.^7^ Furthermore, associations between SIPE and preexisting hypertension, cardiovascular comorbidities, smaller lung volumes, history of SIPE, colonization with respiratory pathogens, use of fish oil/omega 3 fatty acids, low water temperature, long course distance, and overhydration have been suggested.^3^, ^4^, ^5^^,^^7^^,^^9^, ^10^, ^11^, ^12^, ^13^, ^14^ In divers with IPE, psychological stress, wetsuit use, physical exertion, and water aspiration have been proposed as associated factors.^11^^,^^12^^,^^15^

However, knowledge of individual risk factors for developing SIPE during open water swimming remains limited.^14^^,^^16^ Identifying participants at risk of SIPE could enhance individual safety and counseling and provide insights into pathophysiological mechanisms. Vansbrosimningen provides a unique possibility for extending our knowledge of SIPE and the aim of the current study was to investigate individual factors associated with a SIPE diagnosis during the event in 2017 to 2024.

## Study Design and Methods

### Setting

Vansbrosimningen takes place annually in cold-water rivers (16 °C-20 °C) surrounding Vansbro in central Dalecarlia, Sweden; it attracts about 10,000 individuals yearly. In the current study, the participants swum distances of 1,000, 1,500, and 3,000 m in the 2017 to 2019 events, with the addition of 10,000 m in the 2022 to 2024 events. The mean water temperature was 18.1 °C (±1.4 °C) and air temperature 17.4 °C (±2.5 °C) during the event days. The health care organization consisted of a first aid team, ambulances, and a mobile medical unit with physicians and nurses.^6^^,^^7^^,^^17^^,^^18^

### Study Design and Population

In this case-control study, cases were patients diagnosed with SIPE during Vansbrosimningen in 2017 to 2019 and 2022 to 2024 (the event was cancelled in 2020 and 2021 due to the COVID-19 pandemic). Control participants were swimmers aged ≥ 18 years who completed 1,000 to 3,000 m during the 2019 Vansbrosimningen event, without being diagnosed with SIPE. Due to uncertainty regarding whether the event would resume in its original format after the COVID-19 pandemic, control participants were recruited exclusively from the 2019 event.

**Figure 1:**
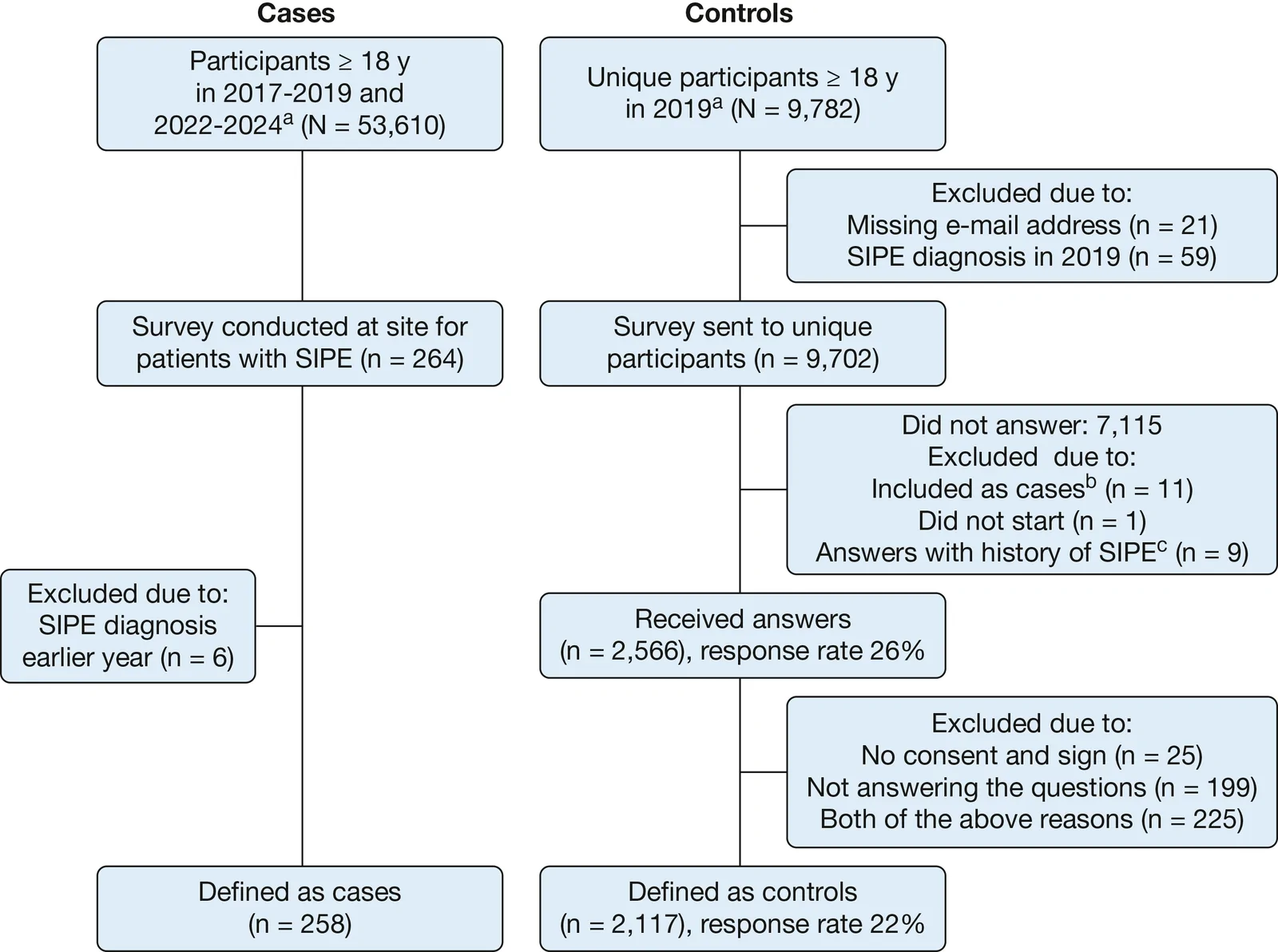
Flowchart inclusion of cases and control participants from Vansbrosimningen. ^a^Included races: Funktionärssimmet, Halvsimmet, Kortsimmet, Tjejsimmet, Öppen älv, and Vansbrosimmet; the 10,000 m race is included for the years 2022 to 2024. ^b^Year 2017, 2018, 2022, 2023, and 2024. ^c^Earlier reported pulmonary edema/SIPE or reported SIPE as the cause of discontinuation of the race. SIPE = swimming-induced pulmonary edema.

Among swimmers (aged ≥ 18 years) who sought medical attention for cough and/or dyspnea, SIPE was diagnosed based on findings of pulmonary edema on lung ultrasound (195 patients).^6^^,^^18^ When lung ultrasound data were not available (predominantly in 2017), the SIPE diagnosis was based on a clinical algorithm incorporating peripheral oxygen saturation ≤ 95% and the presence of crackles on lung auscultation (63 patients).^6^^,^^7^^,^^18^ Patients with repeated SIPE episodes were included only at the first occasion. Control participants were instructed to refer only to the longest race they participated in during 2019, if they had participated in more than 1 race. Control participants who reported respiratory symptoms suggestive of SIPE during Vansbrosimningen or any other swimming event were excluded (Fig 1).

Ethical approvement was received from The Regional Ethical Review Board in Uppsala and The Swedish Ethical Review Authority, Sweden (Dnr 2017/216, 217/216/1, 2017/216/2, 2020-02265, 2021-01708), and all participants gave written informed consent.

### Data Collection

For cases, data on risk factors were collected immediately following the race and during a follow-up telephone interview 10 days later.^7^ For control participants, a web survey was distributed through email, with 5 reminders, available November 4, 2021, to June 30, 2022 (REDCap versions 10.6.9 and 12.0.13). The survey contained the same “multiple choice” questions previously asked to patients, slightly modified to the survey format.^7^ Informed consent was signed digitally. Prior to use, the web survey was evaluated for relevance and simplicity by a group of swimmers and researchers. In addition, 43 patients with SIPE completed the web survey following the follow-up interviews, enabling comparisons of responses (e-Table 1).^19^

Comorbidities of hypertension, asthma, and heart disease were self-reported physician diagnoses, double-checked against reported medication. The following questions were used to assess swimming experience, previous respiratory symptoms, and respiratory tract infection, respectively: “How many times have you practiced swimming in open water this year?” (Never, 1-2, 3-5, > 5 times.) “Have you previously experienced breathing problems or coughing when swimming in open water?” (Yes, No, I have never swum in open water.) “Have you had a respiratory tract infection either during the swim event or within the 7 days preceding it?” (Yes, No, Do not remember.)

Data for attrition analysis for the control group were provided by the Vansbrosimningen office.

### Data Analysis and Models

For a subgroup of cases (n = 43), percent agreement and Cohen’s kappa were used to assess agreement between answers from interviews and the web survey (e-Table 1). For control participants, attrition analysis was performed for nonresponders and responders of the web survey using difference in percentage and the χ^2^ test. Logistic regression was also performed with weighted analyses to account for nonresponder factors in the control group (sex, age, recreational/competitive swimmer, distance, and speed).

For baseline characteristics, continuous data are presented as mean ± SD or as medians with interquartile ranges, and categorical data are presented as numbers with percentages. Univariable and multivariable logistic regression models were used for risk factor analysis, with SIPE diagnosis as a dependent variable.

In the study’s data analysis plan, 2 multivariable logistic regression models were established based on literature, clinical experience, and evaluation according to directed acyclic graphs (e-Fig 1). The first model, focusing on comorbidity and previous swimming experience, included sex, age group (18-30, 31-40, 41-50, 51-60, and ≥ 61 years), hypertension, heart disease, asthma, and frequency of open water swimming during the current season (> 5 times, 3-5 times, 1-2 times, and never).

The second model also included variables for previous respiratory symptoms during swimming and acute respiratory tract infection (e-Fig 1, e-Table 1). The question regarding respiratory tract infections was added to the questionnaire in 2018; therefore, data were missing for 59 participants included in 2017. Also, previous respiratory symptoms during open water swimming may reflect prior SIPE events, potentially associated with other individual risk factors. For respiratory infections, overadjustment could pose a risk when assessing the effect of asthma. Comparison of the 2 regression models, based on data from 2018 to 2024 using the likelihood ratio, statistically favored the second model (*P* < .0001). Areas under the curve were 0.767 and 0.799 for the first and second model, respectively. Multicollinearity was assessed by using an adjusted general variance inflation factor for both models, resulting in general variance inflation factor values of 1.0 to 1.1 for all covariates.

For sensitivity analyses, the second model was repeated with separate additions of height, use of dietary supplements of fish oil/omega 3 fatty acids, before swim warm-up (in water or partially in water), and self-reported psychological stress at start (e-Table 1). These factors were analyzed separately, as the questions regarding fish oil/omega 3 fatty acid use and psychological stress were considered to require cautious interpretation according to results from agreement analysis. Warm-up and physiological stress were added to the survey starting in 2018.

IBM SPSS Statistics for Windows 28.0.0.0 and 29.0.1.0 (IBM SPSS Statistics, IBM Corporation), R version 4.5.2 (R Foundation for Statistical Computing), and GraphPad Prism 10.4.0 (GraphPad Software) were used for statistical analysis and graphic presentation. A *P* value < .05 was considered significant.

## Results

In total, 53,610 adult individuals participated in Vansbrosimningen over the years 2017 to 2019 and 2022 to 2024 (Fig 1). Altogether, 264 cases of SIPE were diagnosed during this period, and of these, 258 unique patients were included as cases in the study.

Of 9,782 unique adult participants in 2019, the survey was emailed to 9,702 swimmers with an available email address (Fig 1). The response rate was 26% (n = 2,566). After further excluding individuals with a history of SIPE and/or those who did not start the race, 2,117 unique participants were included as control participants. Attrition analysis for the web survey showed that responders were more often female, of older age, and with longer swimming distance and higher speed, however with low relative difference between groups (e-Table 2). Analyses with nonresponse weighting accounting for these factors showed higher ORs for all age groups compared with the baseline group (age 18-30 years) in both regression models but did not overall affect the associations with outcome (data not shown).

### Participant Characteristics

Individuals diagnosed with SIPE were more often female and aged > 40 years (Table 1). Overall, participants represent a healthy population. However, a higher percentage of patients with SIPE reported hypertension (11.2% vs 5.4%), heart disease (4.7% vs 0.9%), and/or asthma (15.5% vs 8.8%) compared with the control participants. BMI was similar in both groups, and almost none smoked tobacco.

**Table 1:** Background Characteristics of Participants With SIPE and Control Participants

Characteristic	SIPE (n = 258)	Control Participants (n = 2,117)
Individual characteristics		
Female sex	219 (85)	1,185 (56)^a^
Age group		b
18–30 y	16 (6.2)	356 (17)
31–40 y	38 (15)	423 (20)
41–50 y	92 (36)	611 (29)
51–60 y	73 (28)	561 (27)
≥ 61 y	39 (15)	158 (7.5)
BMI, kg/m^2^	24 (22-27)^b^	24 (23-27)^c^
Tobacco smoking	2 (0.8)	21 (1.0)^a^
Comorbidities and dietary supplements		
Hypertension	29 (11)	114 (5.4)^d^
Heart disease	12 (4.7)	19 (0.9)^a^
Angina, percutaneous coronary intervention with stent/myocardial infarction	2 (0.8)	4 (0.2)
Arrhythmia^e^	7 (2.7)	11 (0.5)
Other	5 (1.9)	2 (0.1)
Asthma	40 (16)	187 (8.8)^c^
Swimming experience and health status at the race		
Frequency of open water swimming during the current season		c
Never	69 (27)	349 (17)
1-2 times	78 (30)	499 (24)
3-5 times	61 (24)	510 (24)
> 5 times	50 (19)	729 (34)
Previous respiratory symptoms during open water swimming	b	f
Yes	69 (27)	240 (11)
No	182 (71)	1,803 (85)
Registration as a recreational swimmer	256 (99)	2,066 (98)
Swimming distance, race time (min)		
1,000 m	69 (48), 34 (31-43)	337 (16), 28 (26-32)
1,500 m	13 (9.1), 62 (49-73)	216 (10), 46 (42-51)
3,000 m	61 (43), 89 (80-105)	1,548 (74), 69 (59-79)
Acute respiratory tract infection^g^	20 (12)^a^	27 (1.3)^h^

Data are presented as No. (%) or median (IQR). IQR = interquartile range; SIPE = swimming-induced pulmonary edema.

a Missing information: n = 10 to 19.

b Missing information: n = 1 to 10.

c Missing information: n = 20 to 29.

d Missing information: n = 50.

e The most common reported arrhythmia was atrial fibrillation in patients diagnosed with SIPE.

f Missing information: n = 74.

g Question included in SIPE patients from year 2018 (n = 199).

h Missing information: n = 63.

Of the 258 patients with SIPE, peripheral oxygen saturation data were available for 255. Among these, 113 patients (44%) had an oxygen saturation ≤ 91%, 89 (35%) between 92% and 95%, and 53 (21%) ≥ 96%. Altogether, 19 patients (7%) with SIPE were referred to the hospital in 2017 to 2024.

### Sex, Age, Comorbidity, and Swimming Experience

**Figure 2:**
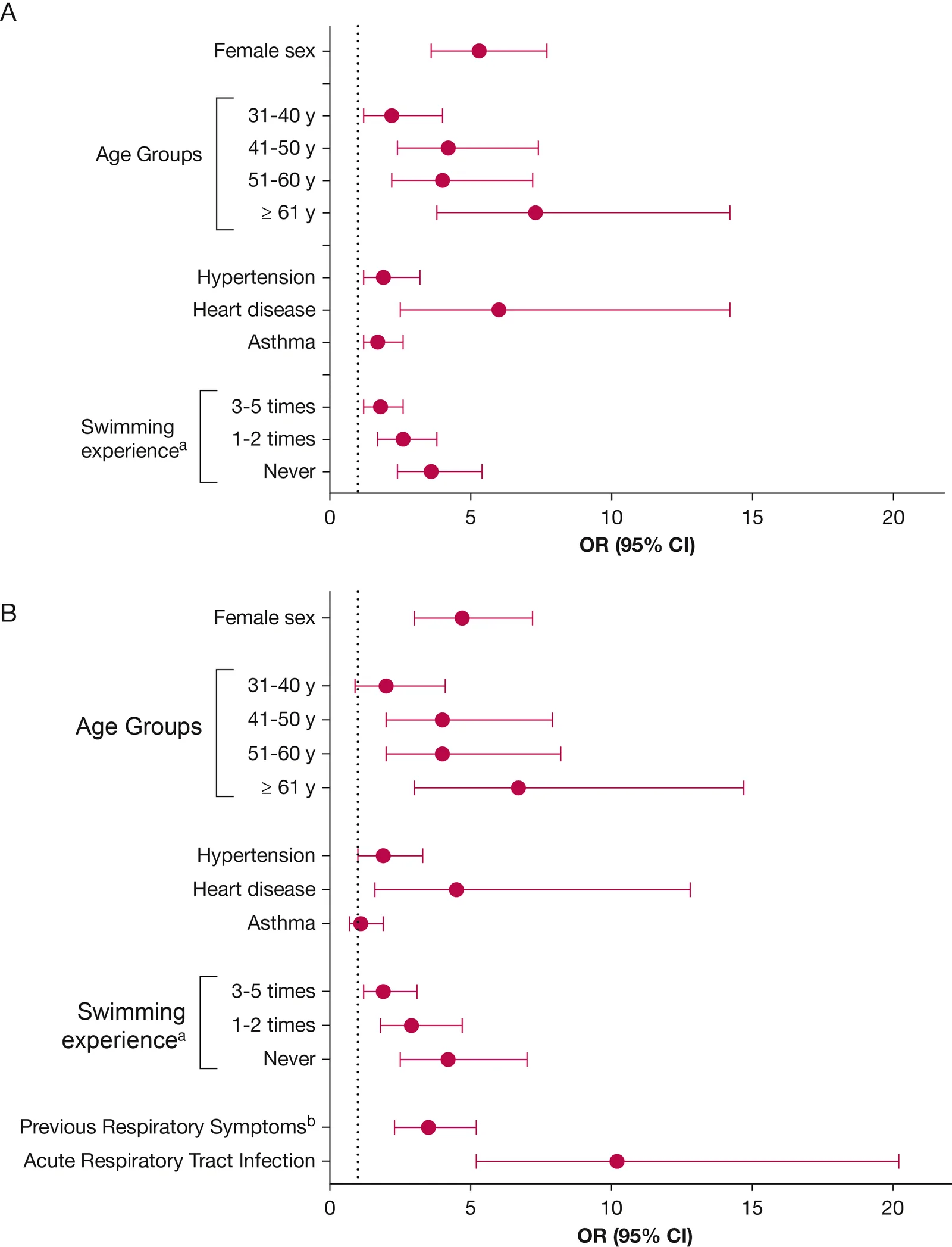
A, B, Forest plot illustrating OR for factors associated with swimming-induced pulmonary edema in adjusted logistic regression from the first model (A) and the second model (B). ^a^Frequency of open water swimming during the current season. ^b^Respiratory symptoms reported in prior open water swimming.

Female sex, higher age, hypertension, heart disease, asthma, and frequency of open water swimming during the current season were all independently associated with SIPE in logistic regression analysis, as shown in the first model (Fig 2A, Table 2). Higher age reflected an increasing risk of SIPE up to the oldest age group (≥ 61 years) compared with the group aged 18 to 20 years. Although the overall incidence of heart disease was low among the participants, it was associated with a high OR for SIPE. Practicing open water swimming prior to the race was protective against SIPE, with the highest risk observed among individuals who had not swum in open water during the current season, with a stepwise lower risk for those who had swum 1 to 2, 3 to 5, and > 5 times, respectively.

**Table 2:** Logistic Regression Analysis of the Association of Sex, Age, Comorbidity With SIPE, and Frequency of Open Water Swimming (First Model)

Variable	Unadjusted Analysis		Adjusted Analysis	
	OR (95% CI)	*P* Value	OR (95% CI)	*P* Value
Sex^a^				
Male	1.0	…		
Female	4.4 (3.1-6.2)	< .001	5.3 (3.6-7.7)	< .001
Age group^b^				
18-30 y	1.0	…		
31-40 y	2.0 (1.1-3.6)	.024	2.2 (1.2-4.0)	.014
41-50 y	3.4 (2.0-5.8)	< .001	4.2 (2.4-7.4)	< .001
51-60 y	2.9 (1.7-5.1)	< .001	4.0 (2.2-7.2)	< .001
≥ 61 y	5.5 (3.0-10.1)	< .001	7.3 (3.8-14.2)	< .001
Hypertension	2.2 (1.4-3.3)	<.001	1.9 (1.2-3.2)	.009
Heart disease	5.3 (2.6-11.1)	< .001	6.0 (2.5-14.2)	< .001
Asthma	1.9 (1.3-2.7)	< .001	1.7 (1.2-2.6)	.007
Frequency of open water swimming during the current season^c^				
> 5 times	1.0	…		
3-5 times	1.7 (1.2-2.6)	.005	1.8 (1.2-2.6)	.007
1-2 times	2.3 (1.6-3.3)	< .001	2.6 (1.7-3.8)	< .001
Never	2.9 (2.0-4.3)	< .001	3.6 (2.4-5.4)	< .001

SIPE = swimming-induced pulmonary edema.

a Male sex used as the reference for sex.

b Age divided into age groups, with the youngest group (18-30 years) used as the reference group.

c Higher swimming frequency used as reference group (> 5 times).

### Previous Respiratory Symptoms and Acute Respiratory Tract Infection

The second logistic regression model also included the following: previous respiratory symptoms during open water swimming and acute respiratory tract infection (Fig 2B, Table 3). Both these factors were significantly associated with SIPE, with the highest risk observed in participants who had an acute respiratory tract infection at the time of the race (OR, 10.2; 95% CI, 5.2-20.2).

**Table 3:** Logistic Regression Analysis of the Association Between SIPE and Sex, Age, Comorbidity, Frequency of Open Water Swimming, Previous Respiratory Symptoms During Open Water Swimming, and Acute Respiratory Tract Infection (Second Model Based on Cases 2018-2024)

Variable	Unadjusted Analysis		Adjusted Analysis: Second Model	
	OR (95% CI)	*P* Value	OR (95% CI)	*P* Value
Sex^a^				
Male	1.0	…		
Female	4.4 (3.1-6.2)	< .001	4.7 (3.0-7.2)	< .001
Age group^b^				
18-30 y	1.0	…		
31-40 y	2.0 (1.1-3.6)	.024	2.0 (0.9-4.1)	.078
41-50 y	3.4 (2.0-5.8)	< .001	4.0 (2.0-7.9)	< .001
51-60 y	2.9 (1.7-5.1)	< .001	4.0 (2.0-8.2)	< .001
≥ 61 y	5.5 (3.0-10.1)	< .001	6.7 (3.0-14.7)	< .001
Hypertension	2.2 (1.4-3.3)	< .001	1.9 (1.0-3.3)	.039
Heart disease	5.3 (2.6-11.1)	< .001	4.5 (1.6-12.8)	.006
Asthma	1.9 (1.3-2.7)	< .001	1.1 (0.7-1.9)	.649
Frequency of open water swimming during the current season^c^				
> 5 times	1.0	…		
3-5 times	1.7 (1.2-2.6)	.005	1.9 (1.2-3.1)	.011
1-2 times	2.3 (1.6-3.3)	< .001	2.9 (1.8-4.7)	< .001
Never	2.9 (2.0-4.3)	< .001	4.2 (2.5-7.0)	< .001
Previous respiratory symptoms during open water swimming	2.8 (2.1-3.9)	< .001	3.5 (2.3-5.2)	< .001
Acute respiratory tract infection^d^	9.0 (5.0-16.5)	<.001	10.2 (5.2-20.2)	< .001

SIPE = swimming-induced pulmonary edema.

a Male sex used as the reference for sex.

b Age divided in age groups, with the youngest group (18-30 years) used as the reference group.

c Higher swimming frequency used as the reference group (> 5 times).

d Measured from year 2018 in patients with SIPE.

When these factors were included in the model, asthma was no longer an independent risk factor for SIPE (Fig 2B, Table 3), even when previous respiratory symptoms during open water swimming or acute respiratory tract infection were added separately (data not shown).

### Other Factors and Sensitivity Analyses

We chose to evaluate a few additional risk factors of interest by unadjusted logistic regression as well as adding them separately to the second regression model. Shorter height was associated with SIPE in unadjusted analysis but not when added to the adjusted model (e-Table 3). Use of dietary supplements of fish oil/omega 3 fatty acids, warming up in water before the swim, and reporting psychological stress at start were also evaluated. None of these factors was associated with SIPE in univariate analysis or in the adjusted model (second model). The use of a wetsuit was not evaluated as virtually all swimmers used wetsuits; 97.7% in patients with SIPE and 97.3% in control participants.

## Discussion

The identification of risk factors for SIPE requires large-scale studies, which is challenging for a condition with a low incidence. We conducted a case-control study based on Sweden’s largest open water swimming event. Previously reported risk factors for SIPE and IPE, including female sex, older age, hypertension, heart disease, respiratory infection, and prior episodes of SIPE, were found to be independently associated with the occurrence of SIPE. We further identified 2 potential risk factors not previously reported in association with SIPE: asthma and limited experience of open water swimming.

The population participating in Vansbrosimningen is overall healthy. We previously presented hypertension and asthma as the most common comorbidities in patients with SIPE.^7^ In the current study, which included a control group of swimmers from the same event, hypertension was associated with SIPE diagnosis together with reported heart disease. In 1989, Wilmshurst et al^5^ described development of hypertension in a group of scuba divers and swimmers with IPE. Hypertension has also been reported in both swimmers and divers with SIPE/IPE.^9^^,^^10^^,^^12^^,^^20^ Hypertension is a well-known risk factor for left ventricular hypertrophy and reduced left ventricular compliance, which may contribute to increased pulmonary capillary pressure during exertion.^9^ Left ventricular hypertrophy has been hypothesized as a marker for SIPE susceptibility in triathletes who died during swimming.^21^ Hypertension is also a known risk factor for atrial fibrillation. Of 12 patients with SIPE reporting heart disease in the current study, 6 reported atrial fibrillation. The strong association observed between heart disease and SIPE in these data is consistent with previous findings in middle-aged divers with IPE.^10^^,^^11^^,^^20^ Studies focusing on underlying cardiac function and dysfunction would be valuable to further clarify the pathophysiology of SIPE and explore the associations with female sex, older age, hypertension, and heart disease.

In a previous follow-up study, we reported an association between asthma and longer symptom duration during SIPE, as well as an increased risk of recurrence.^7^ In the current study, asthma was independently associated with SIPE in the first regression model. However, this association did not persist following adjustment for either previously reported respiratory symptoms during open water swimming or the presence of an acute respiratory tract infection. Adjusting for these 2 variables carries a potential risk of overadjustment. If previously reported respiratory symptoms while swimming in open water represents true SIPE events, adjusting for them may account for the overall individual-level risk for SIPE. For individuals with asthma, respiratory symptoms during physical activity in cold water may reflect asthma-related symptoms. Moreover, asthma may exacerbate the severity and duration of symptoms during a respiratory infection, in which case the infection may act as a mediator between asthma and SIPE. A prevalence of self-reported asthma of 12% was found among participants of 3 Swedish endurance events, including Vansbrosimningen.^22^ In the current study, 16% of patients with SIPE and 9% of control participants reported asthma, compared with 7% to 11% reported for the middle-aged Swedish population.^23^^,^^24^ To our knowledge, there are no previous reports of an association between asthma and developing SIPE/IPE; it would thus be interesting to verify our findings in other cohorts.

As mentioned earlier, both acute respiratory tract infection and previous respiratory symptoms during open water swimming (suggestive of prior SIPE) were independently associated with an increased risk of SIPE. Respiratory tract infection as a risk factor for SIPE is consistent with data from the Navy Sea, Air, and Land (SEAL) candidates, 62% of whom reported preceding respiratory infection symptoms.^13^ In summary, we can speculate that both asthma and respiratory infections increase vulnerability in the small airways and may facilitate the development of pulmonary edema.^25^ Local inflammation compromises the integrity of the alveolar-capillary barrier, making it more susceptible to hydrostatic pressure during exercise and immersion.^26^ These data together emphasize that individuals with an ongoing respiratory infection have a higher risk for SIPE and that open water swimming should be avoided during respiratory infection. Although asthma as a potential risk factor for SIPE warrants further investigation, it is already important to emphasize patient education regarding the acute symptoms of SIPE, particularly among open water swimmers with asthma.

Studies in recreational swimmers are lacking, as most studies of SIPE are published among well-trained militaries and triathletes. Interestingly, the low frequency of open water swimming during the current season turned out to be a risk factor for SIPE. In addition to increased comfort with the open water swimming environment, resulting in reduced psychological stress during the race, we believe that frequent open water practice reflects a higher overall level of swimming experience. Less experienced swimmers exhibit higher oxygen uptake, indicating a greater level of exertion, compared with elite swimmers at the same swimming velocity.^27^ Strenuous exertion has been proposed as a contributing factor involved in the development of IPE.^11^^,^^12^ In fact, patients diagnosed with SIPE in the current cohort swam at slower speeds and were registered for shorter distances compared with the control participants. However, both data are difficult to interpret, as the time in the water may be influenced by experiencing SIPE during the race, and control participants who participated in multiple races were instructed to report the longest race in the web survey.

To account for the potential influence of psychological stress, we included “self-reported stress at start” and “in-water warm-up” in the current sensitivity analyses. Psychological stress might be an interesting factor for SIPE as it can cause, for example, increased sympathetic drive, redistributed blood volume, and changes in breathing patterns.^28^ All of these mechanisms may also be important for SIPE, and psychological stress has been reported in divers with IPE.^11^ Because self-reported psychological stress during the race could be affected by SIPE (eg, symptoms of shortness of breath), we only included stress reported at the start of the analysis. Neither of these factors ultimately was significantly associated with the occurrence of SIPE in the current data.

Because smaller lung volumes have been suggested previously to be associated with SIPE,^4^ we added height to the second model as an indirect measurement for lung capacity. Height was significantly associated with SIPE in univariate analysis, although not in the adjusted model, and it did not affect the other results. These findings suggest that factors other than height and lung volumes may explain the association between female sex, older age, and SIPE. Because Miller et al^9^ surprisingly found that SIPE was associated with the use of fish oil, we were interested in exploring this factor. We could not confirm an association between using dietary supplements of fish oil/omega-3 fatty acids and the occurrence of SIPE.

One strength of this study is that the original cohort of Vansbrosimningen consisted of a large and heterogeneous population, which enhances the generalizability of the findings. Another strength is the low number of SIPE cases lost to follow-up, as well as the use of telephone-based interviews for collection of data for patients with SIPE. As for limitations, the use of a web-based survey for control participants represents a limitation, although this was difficult to avoid. To address this, we performed an agreement analysis comparing responses from telephone interviews vs those obtained from the web-based questionnaire prior to its implementation. To minimize self-reporting bias regarding previous diseases, we requested physician-confirmed medical diagnoses and compared the responses with reported medications. The control group was drawn only from swimmers in 2019 and despite equal incidence of SIPE as well as comorbidity in the patient group across the years, a temporal confounding cannot be excluded. The response rate of 26% for the web survey is comparable to other similar studies.^22^^,^^29^ As suspected, attrition analysis indicated selection bias, with female sex and older age overrepresented among the control participants, although risk estimates remained consistent in weighted regression analyses.^30^

## Interpretation

Consistent with several studies to date, the current data show that female sex, higher age, and hypertension are associated with an increased risk of SIPE. Heart disease, although reported by only a few of the otherwise healthy open water swimmers, was found to be strongly associated with SIPE. Asthma emerged in the current study as a potential risk factor for SIPE, along with ongoing respiratory infection. A compromised alveolar-capillary barrier due to local inflammation may constitute a plausible shared mechanism. Interestingly, engaging in open water swimming prior to the race seemed to be a protective factor against SIPE. Whether this effect reflects a reduction in psychological stress and sympathetic activation or simply serves as a proxy for better swimming technique and experience, cannot be determined based on our current data. Awareness of risk factors for SIPE is important for individual swimmers, as well as organizers of open water swimming events and health care providers.

## Funding/Support

Financial support was provided by the Center for Research and Development, Uppsala University/Region Gävleborg (CFUG), and the 10.13039/100031022Center for Clinical Research (CKF) Dalarna-Uppsala University.

## Financial/Nonfinancial Disclosures

None declared.

## Supplementary Data

e-Online Data

e-Online Data

e-Online Data

e-Online Data
