# Supplementary material for: Suggested Novel Risk Factors for Swimming-Induced Pulmonary Edema: Asthma and Limited Open Water Swimming Experience
Source: Chest. 2026 Mar 26;170(2):477–87. doi: 10.1016/j.chest.2026.02.039 (PMC13494432; doi:10.1016/j.chest.2026.02.039)
Supplement: Supplementary file 1 [file mmc1.pdf]

Supplemental material

Suggested novel risk factors for Swimming-Induced Pulmonary Edema – asthma and limited open water swimming experience

Linda Kristiansson, Claudia Seiler, Annika Braman Eriksson, Josefin Sundh, Maria Hårdstedt.

E-Figure 1

a) *First model* b) *Second model*

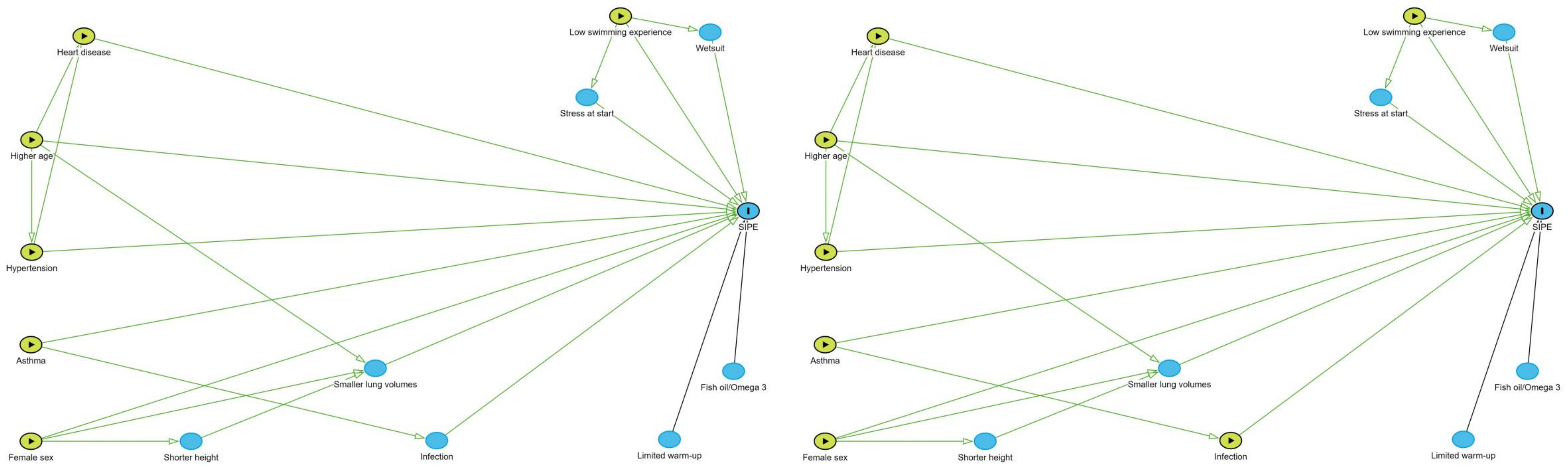

E-Figure 1a, b) Directed acyclic graphs for suspected factors associated with SIPE.
Exposure variables in the *first model*: female sex, higher age, hypertension, heart disease, asthma and low swimming experience. Respiratory tract infection further added to the *second model*. No open biasing path for both models. Previous SIPE not shown in the graph but suspected to affect SIPE and be affected by all factors leading to SIPE. SIPE=swimming-induced pulmonary edema.
