# Supplementary material for: Suggested Novel Risk Factors for Swimming-Induced Pulmonary Edema: Asthma and Limited Open Water Swimming Experience
Source: Chest. 2026 Mar 26;170(2):477–87. doi: 10.1016/j.chest.2026.02.039 (PMC13494432; doi:10.1016/j.chest.2026.02.039)
Supplement: Supplementary file 2 [file mmc2.pdf]

Supplemental material

Suggested novel risk factors for Swimming-Induced Pulmonary Edema – asthma and limited open water swimming experience

E-Table 1

Linda Kristiansson, Claudia Seiler, Annika Braman Eriksson, Josefin Sundh, Maria Hårdstedt.

Supplemental table 1) Agreement analysis between answers at the race and by telephone compared to web-survey

Question	Analysis	Result	Changes for question
Sex	Cohens kappa	0.896	-
Age	Percentage agreement	100%	-
Height*	Weighted kappa	0.975	-
Hypertension	Cohens kappa	1.0	-
Heart disease	Cohens kappa	0.788	-
Asthma	Cohens kappa	0.916	-
Frequency of open water swimming during the present season	Weighted kappa	0.616	Modified**
Previous respiratory symptoms during open water swimming	Cohens kappa	0.588	-
Respiratory tract infection***	Percentage agreement	100%	-
Fishoil/Omega 3	Cohens kappa	0.455	Analyzed with carefulness
Pre-swim warm-up in water or partially in water***	Percentage agreement	100%	-
Stress at start***	Cohens kappa	0.426	Analyzed with carefulness
Wetsuit	Cohens kappa	0.909	-

43/55 SIPE patients answered questions at the race, during telephone follow-up and also the web-survey. The answers from the race and telephone follow-up were compared with the answers from the web-survey. Cohen's kappa and Weighted kappa were interpreted accordning to Landis & Koch (0.41–0.60 = moderate, 0.61–0.75 = substantial, 0.81–1.0 = almost perfect). *Height compared in three categories. **The web survey clarified that the season ran from January to the July race. ***Question included from the year 2018. SIPE = swimming-induced pulmonary edema
