# Supplementary material for: Suggested Novel Risk Factors for Swimming-Induced Pulmonary Edema: Asthma and Limited Open Water Swimming Experience
Source: Chest. 2026 Mar 26;170(2):477–87. doi: 10.1016/j.chest.2026.02.039 (PMC13494432; doi:10.1016/j.chest.2026.02.039)
Supplement: Supplementary file 3 [file mmc3.pdf]

Supplemental material

Suggested novel risk factors for Swimming-Induced Pulmonary Edema – asthma and limited open water swimming experience

Linda Kristiansson, Claudia Seiler, Annika Braman Eriksson, Josefin Sundh, Maria Hårdstedt.

E-table 2

Supplemental table 2) Attrition analysis in the group receiving the survey (n =9681[a])

	Non-responders[b] (n =7564)	Responders (n =2117)	Difference	P-value[c]
Sex:				
Female; n (%)	3878 (51)	1182 (56)	4,6%	<0.001
Male; n (%)	3686 (49)	935 (44)	4,6%	
Age group:				
18-30 y	2276 (30)	369 (17)	12,7%	<0.001
31-40 y	1859 (25)	424 (20)	4,5%	
41-50 y	1773 (23)	612 (29)	5,5%	
51-60 y	1320 (17)	556 (26)	8,8%	
≥61 y	336 (4)	156 (7)	2,9%	
Registered for distance; n (%)				
1000 m	1556 (21)	344 (16)	4,3%	<0.001
1500 m	799 (11)	223 (11)	0,0%	
3000 m	5209 (69)	1550 (73)	4,4%	
Registration as recreational or competitive swimmer; n (%)				
Recreational	7430 (98)	2066 (98)	0,6%	0.058
Competitive	134 (2)	51 (2)	0,6%	
Did not finish; n (%)	44 (1)	5 (0)	0,3%	0.048
Complete the race; n (%)	7520 (99)	2112 (100)	0,3%	
Completed race with pace ≥20 minutes/km	6386 (85)	1708 (81)	4,0%	<0.001
Completed race with pace <20 minutes/km	1134 (15)	404 (19)	4,0%	

[a]Excluded in non-response analysis: SIPE patients included as cases, did not started and/or answers with history of SIPE (n =21). [b]Swimmers who did not answer or who did not give consent to and sign the survey are considered as non-responders. [c]Chi-square Test used for analysis. SIPE=Swimming-induced pulmonary edema
