# Supplementary material for: Suggested Novel Risk Factors for Swimming-Induced Pulmonary Edema: Asthma and Limited Open Water Swimming Experience
Source: Chest. 2026 Mar 26;170(2):477–87. doi: 10.1016/j.chest.2026.02.039 (PMC13494432; doi:10.1016/j.chest.2026.02.039)
Supplement: Supplementary file 4 [file mmc4.pdf]

Supplemental material

Suggested novel risk factors for Swimming-Induced Pulmonary Edema – asthma and limited open water swimming experience

Linda Kristiansson, Claudia Seiler, Annika Braman Eriksson, Josefin Sundh, Maria Hårdstedt.

E-table 3

e-Table 3) Sensitivity analyses for the second model

	**SIPE (n =258) (%)**	**Controls (n =2117)**	**Unadjusted analysis**		**Adjusted analysis - second model**	
**Variable**			OR (95% CI)	p-value	OR (95% CI)	p-value
**Height***	[a]	[b]				
**180-204 cm**	190 (73.6%)	1091 (51.5%)	1.0	...		
**175-179 cm**	32 (12.4%)	365 (17.2 %)	1.8 (1.1 - 3.0)	0.025	0.9 (0.5 - 1.8)	0.907
**147-174 cm**	31 (12.0%)	633 (29.9%)	3.6 (2.4 - 5.3)	<.001	1.0 (0.6 - 1.9)	0.921
**Use of fish oil/Omega 3**	28[c] (10.9%)	170[d] (8.0%)	1.4 (0.9 - 2.2)	0.100	1.2 (0.7 - 2.1)	0.563
**Pre-swim warm-up in water or partially in water****	9[e] (4.5%)	109 (1.3%)	0.9 (0.5 - 1.8)	0.780	0.6 (0.3 - 1.3)	0.196
**Stress at start**	51 (57.3%)	282[a] (67.0%)	0.7 (0.4 - 1.0)	0.078	0.8 (0.5 - 1.4)	0.419

All factors separately added to the second model. In the second model, logistic regression analysis included following factors: sex, age, co-morbidity, previous swimming experience in open water, previous respiratory symptoms in open water and acute respiratory tract infection. *Height divided into groups with the highest group (180-204 cm) used as the reference. **Compared to only warm-up at land. Missing information: [a]n=1-10, [b]n=20-29, [c]n=10-19, [d]n=70-79, [e]n=60-69.
